# Brain-Derived Neurotrophic Factor-Dependent cdk1 Inhibition Prevents G2/M Progression in Differentiating Tetraploid Neurons

**DOI:** 10.1371/journal.pone.0064890

**Published:** 2013-05-31

**Authors:** María C. Ovejero-Benito, José M. Frade

**Affiliations:** Department of Molecular, Cellular, and Developmental Neurobiology, Instituto Cajal, Consejo Superior de Investigaciones Científicas, Madrid, Spain; Instituto de Neurociencias, CSIC and UMH, Spain

## Abstract

Neurodegeneration is often associated with DNA synthesis in neurons, the latter usually remaining for a long time as tetraploid cells before dying by apoptosis. The molecular mechanism preventing G2/M transition in these neurons remains unknown, but it may be reminiscent of the mechanism that maintains tetraploid retinal ganglion cells (RGCs) in a G2-like state during normal development, thus preventing their death. Here we show that this latter process, known to depend on brain-derived neurotrophic factor (BDNF), requires the inhibition of cdk1 by TrkB. We demonstrate that a subpopulation of chick RGCs previously shown to become tetraploid co-expresses TrkB and cdk1 in vivo. By using an in vitro system that recapitulates differentiation and cell cycle re-entry of chick retinal neurons we show that BDNF, employed at concentrations specific for the TrkB receptor, reduces the expression of cdk1 in TrkB-positive, differentiating neurons. In this system, BDNF also inhibits the activity of both endogenous cdk1 and exogenously-expressed cdk1/cyclin B1 complex. This inhibition correlates with the phosphorylation of cdk1 at Tyr15, an effect that can be prevented with K252a, a tyrosine kinase inhibitor commonly used to prevent the activity of neurotrophins through their Trk receptors. The effect of BDNF on cdk1 activity is Tyr15-specific since BDNF cannot prevent the activity of a constitutively active form of cdk1 (Tyr15Phe) when expressed in differentiating retinal neurons. We also show that BDNF-dependent phosphorylation of cdk1 at Tyr15 could not be blocked with MK-1775, a Wee1-selective inhibitor, indicating that Tyr15 phosphorylation in cdk1 does not seem to occur through the canonical mechanism observed in proliferating cells. We conclude that the inhibition of both expression and activity of cdk1 through a BDNF-dependent mechanism contributes to the maintenance of tetraploid RGCs in a G2-like state.

## Introduction

Reactivation of cell cycle and DNA synthesis in neurons represents a common feature of certain neuropathological states [1], including Alzheimer’s disease (AD) and ischemia/hypoxia [2]–[5]. Interestingly, neurons that duplicate their DNA are rarely observed to undergo mitosis, and they remain for long time with double the normal amount of DNA in their nuclei before dying by apoptosis [5], [6]. In contrast to the enormous effort made by several laboratories during the last decade to study the molecular basis for neuronal cell cycle reactivation [7]–[12], the mechanism used by neurons to prevent G2/M progression once that the cell cycle has been reactivated is basically unknown [5]. The understanding of this mechanism could facilitate the development of novel approaches to avoid aberrant mitotic events in pathologically-generated tetraploid neurons [13], [14], thus facilitating their survival.

We have previously demonstrated that the neurotrophin nerve growth factor (NGF), acting through the common p75 neurotrophin receptor (p75^NTR^), induces cell cycle reactivation in a small population of chick differentiating retinal ganglion cells (RGCs). Cell cycle re-entry in these neurons occurs as they migrate from the apical portion of the neuroepithelium, where they are born, to the basal neuroepithelium, where the ganglion cell layer (GCL) arises [15]. These neurons are known to express E2F1 and E2F4 in the absence of retinoblastoma protein (Rb) and, after DNA duplication, they remain in a G2-like state in the GCL [12], [15]. The mechanism preventing G2/M transition in differentiating RGCs that duplicate their DNA depends on the presence of endogenous brain-derived neurotrophic factor (BDNF) [15], which is known to be expressed by the pigment epithelium that surrounds the retina, and the retina itself [16]. In the absence of BDNF, differentiating tetraploid RGCs upregulate cyclin B2 expression, undergo G2/M transition, and die by apoptosis [15]–[19], a process that can be blocked with cyclin-dependent kinase (cdk) inhibitors [17]. Cell cycle reentry in differentiating RGCs and maintenance of these cells in a G2-like state can be considered as part of a physiological process taking place in the developing nervous system aimed at inducing somatic tetraploidy in specific neuronal types [15], [20], [21]. Overall, these observations are compatible with BDNF being also responsible for the maintenance in a G2-like state of pathologically-generated tetraploid neurons, thus preventing their death [22].

Neurotrophins, including NGF, BDNF, neurotrophin-3 (NT3) and NT4/5, are trophic factors with multiple functions in both the developing and adult nervous system [23]. These factors are known to transduce their signals through two different types of receptors: p75^NTR^ and the members of the Trk family of receptor tyrosine kinases [24]. While p75^NTR^ can be activated with low affinity by all neurotrophins, signaling of each of the four mammalian neurotrophins can also be mediated through activation of one of the three members of the Trk family: TrkA, TrkB, and TrkC, which are high affinity receptors for NGF, BDNF/NT4, and NT3, respectively [23], [24].

G2/M transition in proliferating cells is regulated by cdk1 [25], suggesting that this cyclin B-dependent kinase may play an important role in BDNF-dependent G2/M arrest. In this study we show that TrkB is expressed in a subpopulation of differentiating RGCs susceptible to become tetraploid in vivo. We also show that cdk1 colocalizes with TrkB in these neurons, and that BDNF, likely acting through the neurotrophin receptor TrkB, decreases the expression and activity of cdk1 in differentiating chick retinal neurons (DCRNs) that have reactivated the cell cycle in response to NGF. We also provide evidence that the mechanism used by BDNF to prevent cdk1 activity in NGF-treated DCRNs is likely based on TrkB-dependent, Wee1-independent Tyr15 phosphorylation of cdk1.

## Materials and Methods

### Ethics Statement

This study was carried out in strict accordance with the guidelines of the European Union Directive 2010/63/EU on the protection of animals used for scientific purposes. Ethical approval for this study is not mandatory since it only employed chick embryos from the first two thirds of their development. In any case, experimental procedures were included in a Project approved by the Committee on the Ethics of Animal Experiments of the CSIC (BFU2009-07671).

### Chick Embryos

Fertilized eggs from White Leghorn hens were obtained from a local supplier (Granja Santa Isabel, Spain). They were incubated at 38.5°C in an atmosphere of 70% humidity. The embryos were staged according to [26].

### Primary Antibodies

The mouse mAb [A17] to cdk1 (Abcam) was raised against residues 220–227 of Xenopus cdk1 (accession number: NP_001080554) [27]. This sequence (LGTPNNEV), contained within the C-terminal region of the molecule, is functionally conserved in chick cdk1 but not in other cdks [27], [28]. This antibody was used at 3.75 µg/ml for immunoprecipitation, at 4 µg/ml for sandwich enzyme-linked immunosorbent assay (ELISA) plate coating, and diluted 1/100 for immunohistochemistry. The mouse anti-cdk1 p34 (17) mAb (Santa Cruz Biotechnology) recognizes the sequence flanked by amino acids 224–230 of human origin (NNEVWPE; accession number: NP_001777) which is functionally conserved with the chick sequence. This antibody was used at 1/2,000 dilution for immunohistochemistry. The mouse mAb anti-cdk1 p34 (B-6) (Santa Cruz Biotechnology) is specific for an epitope mapping between amino acids 2–30 near the N-terminus of human cdk1 (EDYTKIEKIGEGTYGVVYKGRHKTTGQVVA; accession number: NP_001777), which is fully conserved with the chick cdk1 sequence. This antibody was used at 1/100 dilution for immunohistochemistry, and at 1/10,000 dilution for western blot. The rabbit polyclonal anti-cdk1 (pTyr15) antibody (AnaSpec) specifically recognizes cdk1 phosphorylated in Tyr15 of zebrafish, chicken, mouse, and human origin. This antibody was used at 1.5 µg/ml for sandwich ELISA. The rabbit polyclonal antibody against the PSTAIRE domain of cdk1 (Santa Cruz Biotechnology) was used at 1/300 dilution for sandwich ELISA, and diluted 1/2,000 for western blot. The rabbit anti-phospho-Rb (Ser780) antibody (HTScan CDK1/CycB Kinase Assay Kit; Cell Signaling Technology) was used at 1/1,000 dilution for sandwich ELISA. The rabbit polyclonal antiserum [9650] against the extracellular domain of human p75^NTR^ was a kind gift of Moses V. Chao (New York University), and it was diluted 1/1,000 for immunohistochemistry. The mouse polyclonal antibody against p75^NTR^ [29], kindly provided by Alfredo Rodríguez-Tébar (CABIMER, Seville, Spain), was diluted at 1/500 for immunohistochemistry. The rabbit polyclonal antiserum against the extracellular domain of chick TrkB was a generous gift from Louis F. Reichardt (University of California, San Francisco), and it was used at 1/2,000 dilution for immunohistochemistry. The anti-Rb mAb G3-245 (BD Biosciences Pharmingen) was used at 1/400 dilution for immunohistochemistry. This antibody recognizes an epitope between amino acids 332-344 of the human Rb protein (accession number: NP_000312), which is conserved in avian Rb. The anti-phosphoHistone H3 rabbit polyclonal antiserum (Upstate Biotechnology) was diluted 1/1,000 for immunohistochemistry and 1/500 for immunocytochemistry. The mouse mAb against neuron-specific βIII tubulin (clone 5G8; MILLIPORE/Chemicon) specifically recognizes RGCs from early stages of differentiation [30] and it was diluted 1/1,000 for immunohistochemistry. The rabbit anti-GFP antiserum (Invitrogen) was used at 1/1,000 dilution for immunocytochemistry. The mouse mAb against β-actin (Sigma) was used at 1/20,000 for western blot.

### Plasmids

The cdk1 and cyclin B1 expression vectors, previously described by [31], were a gift from Gavin Brooks (The University of Reading, UK). A vector expressing a constitutively active, mutant form of cdk1 in which Tyr15 has been substituted by Phe [32] was a gift from Ruth J. Muschel (University of Oxford, UK). In this construct, the mutant form of cdk1 is fused to EGFP. The pEGFP-N1 plasmid (BD Biosciences Clontech) was used as a control vector.

### Immunostaining

Immunocytochemistry was performed in cells fixed for 15 min with 4% paraformaldehyde at room temperature (RT), and permeabilized for 30 min with phosphate buffered saline (PBS) containing 0.05% Triton X-100 (Sigma) (PBTx) and 10% Fetal Calf Serum (FCS; Invitrogen). The cells were then incubated for 2 h (at RT) or overnight (ON) (at 4°C) with PBTx containing 1% FCS and the appropriate primary antibody. Following five washes in PBTx, the cells were incubated for an additional 1 h in PBTx containing 1% FCS and a 1/1,000 dilution of Cy2-conjugated Affinipure Goat Anti-Rabbit IgG (H+L) (Jackson Immunoresearch). Nuclear labeling was then performed with PBS containing 1 µg/ml bisbenzimide, and the preparations were then mounted in glycerol (Panreac)/PBS (1∶1).

For immunohistochemistry, embryos were fixed for 8 h at 4°C with 4% paraformaldehyde (Merck), cryopreserved ON at 4°C in PBS containing 30% sucrose (Merck), and embedded in the OCT compound Tissue-Tek (Sakura). Cryosections (12 µm) were permeabilized and blocked for 30 minutes at RT in 0.5% PBTx and 10% FCS, and they were then incubated ON at 4°C with the primary antibodies in PBTx plus 1% FCS. After 5 washes with PBTx, the sections were incubated for 1h at RT with an Alexa 594-coupled anti-mouse IgG antibody (Invitrogen) and Cy2-conjugated anti-rabbit IgG (H+L) antibody (Jackson Immunoresearch), each diluted 1/1,000. The sections were finally washed 5 times in PBTx, once in PBS, and they were then incubated with 1 µg/ml bisbenzimide (Sigma) in PBS before mounting in glycerol/PBS (1∶1). Negative controls performed using only secondary antibodies resulted in lack of specific immunostaining (data not shown).

### Explant Electroporation

Electroporation of retinal explants was performed as described previously [15]. Briefly, E6 chick retinas were dissected away from the pigment epithelium and fragmented into small pieces of around 10 mm^2^. These retinal fragments were laid onto a glass coverslips and subsequently immersed in 4 µl PBS containing different plasmid combinations. Electroporation was performed with four 50 milliseconds pulses of 25–27 V, at a 200 milliseconds frequency. After electroporation, the explants were grown in suspension for 3–4 h in 50% Dulbecco-Modified Eagle Medium (DMEM)/50% F12 HAM (Sigma) with N2 supplement (Sigma) (DMEM/F12/N2). Explants were then collected, dissociated, and cultured as described below. To facilitate EGFP visualization, cells were immunostained with and anti-GFP antibody. As an average, 0.5–1% of DCRNs were observed to express GFP in these cultures.

### Cell Culture

Chicken embryonic fibroblast (CEFs) cultures were established from E9 chicken embryos, which were decapitated and eviscerated. The remaining tissue was minced and trypsinized for 30 min at 37°C with a 0,25% solution of trypsin (Worthington) in PBS. The trypsin was then inactivated with 10% FCS, and the tissue was dissociated by gentle trituration, centrifuged at 300×g for 10 min, and plated in the DMEM/10% FCS containing 25 U/ml penicillin and 25 µg/ml streptamicin (Invitrogen). Experiments were performed with CEFs at 25–30% confluence. Neuronal precursors isolated from the E6 chick retina are susceptible to differentiate in the presence of laminin-1 and insulin [33], [34], the latter present in the N2 supplement, giving rise to DCRNs. Exogenous NGF can induce cell cycle re-entry and apoptosis of DCRNs through p75^NTR^ [17], [35]. Retinal neurogenic cultures were performed as previously described [36]. Briefly, dissociated retinal cells were suspended in DMEM/F12/N2, and plated at a density of 10,000–100.000 cells/cm^2^ on 10-mm round glass coverslips previously coated with 500 µg/ml poly (D-L) ornithine (Sigma) and 10 µg/ml laminin-1 (natural mouse laminin; Invitrogen). Cultures were maintained ON in four-well dishes (Greiner Bio-One, Germany). Alternatively, E6 retinal cells were cultured on either P100 tissue culture dishes (8–10×10^6^ cells/plate) or P35 tissue culture dishes (1.5×10^6^ cells/plate), previously treated with poly (D-L) ornithine and laminin-1, as above. Cells were cultured in DMEM/F12/N2. Cultures were maintained at 37°C for 20 h in a water-saturated atmosphere containing 5% CO_2_ and where stated, they were supplemented with recombinant NGF (Alomone Labs, and Sigma) and/or BDNF (Alomone Labs) for different time periods. NGF was added at the time of plating at either 1 ng/ml (specific for TrkA binding [37]) or 100 ng/ml (saturating concentration for p75^NTR^ binding [17]), and BDNF was added either at the time of plating or 30 min before processing at 2 ng/ml, a concentration specific for TrkB [38]. Nocodazole (Sigma) was prepared in dimethyl sulfoxide (Sigma) and used at 0.4 µg/ml for G2/M transition arrest. K252a (200 nM) (Alomone Labs) and MK-1775 (300 nM) (Axon MedChem), both prepared in dimethyl sulfoxide, were added to the culture medium 10 min before BDNF treatment (DCRNs). CEFs were treated for 20 h with 300 nM MK-1775.

### Reverse Transcriptase-Polymerase Chain Reaction (RT-PCR)

mRNA from DCRNs maintained for 20 h under control conditions or in the presence of BDNF was extracted using the QuickPrep Micro mRNA purification kit (GE Healthcare). cDNA was then prepared using the First-Strand cDNA synthesis kit (GE Healthcare). PCR amplification was performed using standard procedures. The PCR primers used for chick *Cdk1* correspond to bp 93–112 and complementary to bp 540–559 (accession number NM_205314). PCR primers for chick *Gapdh* correspond to bp 944–963 and complementary to bp 1,219–1,238 (accession number K01458). *Cdk1* was amplified for 31–40 cycles, whereas *Gapdh* was amplified for 22–31 cycles. Under these conditions, amplification was linear. Band densities were quantified using ImageJ software after subtracting the background. The *Cdk1*/*Gapdh* ratio was estimated from 4 independent experiments. No amplification products were obtained in reactions lacking reverse transcriptase.

### Cell Extracts

Cultures of dissociated E6 retinal cells were placed on ice, washed with ice-cold PBS, and incubated for 30 minutes with 150 µl (for western blot) or 700 µl (for immunoprecipitation) lysis buffer containing 50 mM Tris-HCl pH 8.0, 150 mM NaCl, 1% Triton X-100, 1× protease inhibitor cocktail (Roche), and 1× phosphatase inhibitor cocktail 1 (Sigma, only for sandwich ELISA). Cell lysates were scraped with a rubber policeman, and those used for western blot were further homogenized by passage through a 22 ga needle (5 times). Extracts were then centrifuged at 13,000 × g for 10 minutes at 4°C and the supernatant was either directly used for immunoprecipitation or mixed with 1 volume of 2× Laemli’s buffer and boiled for 5 minutes (western blot). For sandwich ELISA, cultures were incubated for 30 minutes with 600 µl hypotonic buffer (20 mM Tris-HCl pH 7.4; 10 mM NaCl), scraped with a rubber policeman, and extracted using a potter homogenizer. Lysates were centrifuged at 16,000 × g for 10 minutes.

### Immunoprecipitation

Cell extracts derived from 10^7^ cells were obtained as described above, and 50 µl of the supernatant from each extract was mixed with 50 µl 2× Laemli’s buffer and boiled for 5 minutes (Input samples). The rest of the supernatant was precleared for 1 h at 4°C with 20 µl (bed volume) nProtein A Sepharose (GE Healthcare), and then incubated with the anti-cdk1 mAb A17 ON at 4°C followed by incubation with 20 µl (bed volume) nProtein A Sepharose for 1 h at 4°C. Immunoprecipitates were washed five times with 1 ml lysis buffer, and resuspended with 2× kinase assay buffer (see below).

### Cdk1 Activity Assay

Cdk1 activity was measured in cell extracts from E6 retinal cells (see above), following a method based on the immunokinase assays described by [39]. Briefly, cdk1 was immunoprecipitated using 25 µl (bed volume) nProtein A Sepharose with the A17 mAb as described by [39]. In addition, a specificity control was performed with cell extracts four-fold concentrated using Amicon Ultra 3K centrifugal filter (Millipore). 25 µl of either immunoprecipitates or concentrated extracts were mixed with 25 µl 2× kinase assay buffer (HTScan CDK1/CycB Kinase Assay Kit; Cell Signaling Technology). These mixes were incubated for 30 min with 25µl 2× ATP substrate cocktail containing a Rb (Ser780) biotinylated peptide (HTScan CDK1/CycB Kinase Assay Kit; Cell Signaling Technology), which is known to be phosphorylated in vitro by different cdks, including cdk1, cdk2, and cdk4 [40]. The reaction was stopped with 50 µl 50 mM EDTA pH 8.0, and then centrifuged for 10 min at 16,000 × g. The supernatants were diluted with distilled water (1∶3) and triplicates (25 µl each) were incubated for 2 h at RT in Reacti-Bind Streptavidin Coated 96-Well Plates (Pierce). Wells were washed three times with PBS/0.1% Tween 20 (PBTw), and then incubated ON at 4°C with anti-phosphoRb (Ser780) in PBTw containing 1% bovine serum albumin (BSA). Wells were then washed three times with PBTw, and then incubated for 2h at RT with the Peroxidase-conjugated Affinipure goat anti-rabbit IgG antibody (Jackson Immunoresearch) diluted 1/5000 in PBTw containing 1% BSA. Finally, wells were washed three times with PBTw, and incubated for 15–30 min at RT with 2,2'-Azino-di-[3-ethylbenzthiazoline sulfonate (6)] diammonium salt (ABTS; Roche), before measuring the green intensity level with a Thermo Labsystems Multiskan Ascent Photometric plate reader (Labsystems Multiskan Ascent) at a wavelength of 405 nm. Cdk1 activity for each experimental condition was normalized to the levels of cdk1 as measured by western blot analysis from the input samples.

### Western Blot

Cell extracts obtained as described above (corresponding to 1.5×10^6^ E6 retinal cells) were separated by SDS PAGE on 12% acrylamide gels and transferred to Immun-Blot PVDF membranes (BioRad). The membranes were incubated for 1 h with 2% ECL *Advance* blocking agent (ECL Advanced Western Blotting Detection Kit; GE Healthcare) in PBTw, and incubated ON at RT with the appropriate antisera in blocking agent. After washing the membranes five times in PBTw, they were incubated for 1 h at RT with a 1/1,600,000 dilution Peroxidase-conjugated Affinipure goat anti-rabbit IgG antibody (Jackson Immunoresearch), or a 1/500,000 dilution of goat anti-mouse IgG Horseradish Peroxidase Conjugate antibody (BioRad) in blocking agent. Finally, they were washed again as above and the protein bands were visualized using ECL Advanced Western Blotting Detection Kit. Band densities were determined using ImageJ software after subtracting the background.

### Sandwich ELISA

ELISA plates were coated ON at 4°C with anti-cdk1 mAb A17 in PBS. After two washes with PBS, unspecific binding was blocked by incubating the wells for 3 h at RT with 250 µl PBS containing 3% BSA. Then, wells were washed twice with PBS and incubated for at least 4 h at RT with 50 µl cell extracts (corresponding to 6.5×10^5^ E6 retinal cells) obtained as described above. After washing twice with PBS, wells were incubated ON at 4°C with 50 µl PBS/3% BSA containing either anti-cdk1 (pTyr15) antibody or anti-cdk1 PSTAIRE antibody. Afterwards, wells were washed four times with PBS and incubated for 2 h at RT with 50 µl of a 1/5,000 dilution of Peroxidase-conjugated Affinipure goat anti-rabbit IgG antibody (Jackson Immunoresearch) in PBS/3% BSA. After washing four times with PBS, wells were incubated for 15–60 minutes with 100 µl ABTS before measuring the green intensity level with a Multiskan Ascent Photometric plate reader (Thermo Scientific) at a wavelength of 405 nm. Final values (in triplicate) were obtained by subtracting the average background. The sandwich ELISA assay was validated using a human cdk1 recombinant protein with a glutation S-transferase (GST) tag at its N-terminus (Abnova), as compared with a previously described GST recombinant protein [41]. The capacity of the sandwich ELISA assay to detect Tyr15-phosphorylated cdk1 was confirmed using cell lysates from NGF/BDNF-treated DCRNs incubated for 1 h at 37°C with (or without) 1 unit of calf intestine alkaline phosphatase (CIP; Fermentas) per µg of protein. Reactions were performed in the buffer provided by the manufacturer.

### Cell Counting

Cells were counted on a Nikon (Melville, NY) E80i microscope using a 40× objective with phase contrast and epifluorescence illumination, and an average of 100–400 cells were analyzed per coverslip. Mitotic nuclei were identified by either DNA staining with 1 µg/ml bisbenzimide (Sigma) or phosphoHistone H3 immunostaining. Apoptosis was measured as the percentage of cells showing pyknotic nuclei, as evidenced by bisbenzimide staining.

### Statistical Analyses

Quantitative data are represented as the mean ± SEM. Statistical differences were analyzed using either Student’s *t*-test or ANOVA.

## Results

### TrkB is Expressed by a Subset of Differentiating RGCs Susceptible to become Tetraploid in vivo

**Figure 1:**
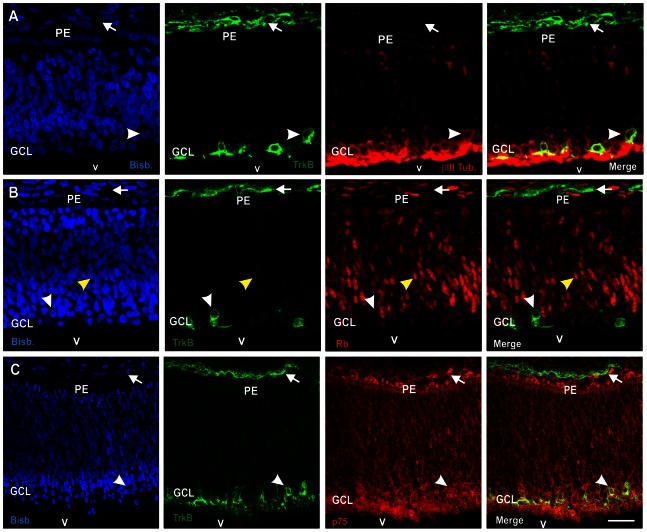
TrkB is expressed by a subset of RGCs lacking Rb expression. Confocal sections from retinas of E6 chick embryos were double labeled with an anti-TrkB specific antiserum (green) and anti-βIII tubulin (βIII Tub.) (**A**), anti-Rb (**B**), or anti-p75^NTR^ (p75) (**C**) antibodies (red). Nuclear staining with bisbenzimide is shown in blue (Bisb.). (**A**) TrkB-positive cells colocalize with βIII tubulin in the basal retina, close to the presumptive GCL (arrowhead). (**B**) TrkB-positive cells do not express Rb (arrowhead). Yellow arrowhead: a cell expressing Rb. (**C**) TrkB-positive cells colocalize with p75^NTR^ (arrowhead). Arrow: TrkB-positive cells surrounding the eye. GCL: ganglion cell layer; PE: pigment epithelium; v: vitreous body. Bar: 20 µm (**A**,**B**), 40 µm (**C**).

To verify whether the BDNF neurotrophic receptor TrkB is expressed by the RGCs in vivo during the period of neuronal tetraploidization [15], E6 chick embryos were fixed and dorso-ventral cryosections containing the central retina were subjected to immunohistochemistry with a TrkB-specific antibody. In the E6 embryonic retina, TrkB-specific immunoreactivity was observed in a subpopulation of differentiating RGCs, as defined by the expression of βIII tubulin and their localization close to the vitreal surface, where the prospective GCL resides (Fig. 1A). Most of the TrkB-positive RGCs were observed to lack Rb expression (69 out of 86 neurons, n = 2 embryos), and those positive for Rb expressed it at extremely low levels as compared with precursor cells (Fig. 1B). This suggests that TrkB is preferentially expressed by differentiating RGCs that reactivate the cell cycle to become tetraploid, known to lack Rb expression and depend on BDNF to prevent G2/M transition [15]. Moreover, double immunolabeling indicated that all TrkB-positive RGCs analyzed express p75^NTR^ (104 out of 104 neurons, n = 2 embryos) (Fig. 1C), a receptor present in all differentiating RGCs, which has been shown to induce cell cycle reactivation in those RGCs lacking Rb expression [15].

### Cdk1 is Expressed by Differentiating RGCs Undergoing Ectopic Mitoses in vivo

**Figure 2:**
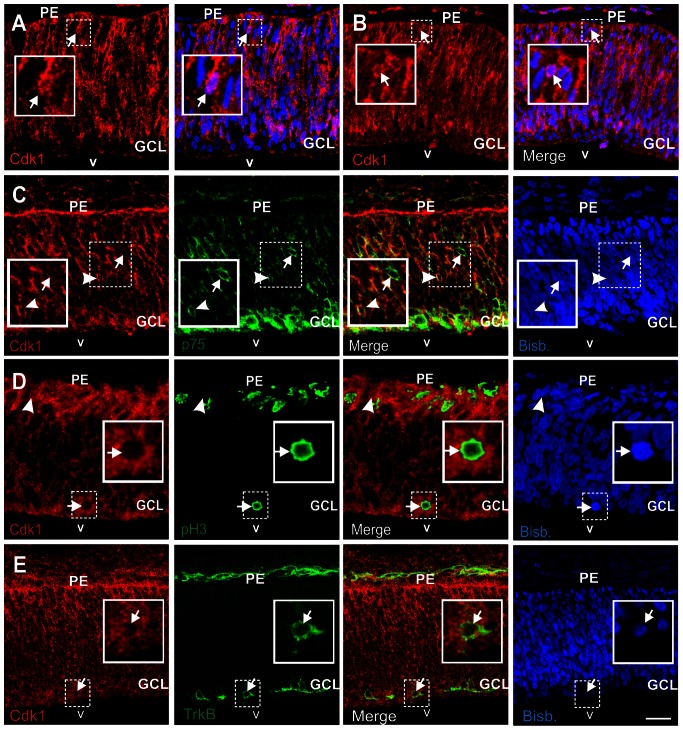
Cdk1 is expressed by p75^NTR^- and TrkB-positive DCRNs and colocalizes with basally-located mitosis. Confocal sections from retinas of E6 chick embryos were double labeled with the anti-cdk1 specific mAbs (red) (B-6) (**A**), [A17] (**B**), and (17) (**C**-**E**), as well as with anti-p75^NTR^ (p75) (**C**), anti-phosphoHistone H3 (pH3) (**D**), or anti-TrkB (**E**) antibodies (green). Nuclear staining with bisbenzimide is shown in blue (Bisb.). (**A**,**B**) Cdk1 immunostaining is observed in the cytoplasm of a subpopulation of retinal cells and often in nuclei located apically (arrows). (**C**) A subset of cdk1-positive cells co-localize with p75^NTR^ (arrowhead), whereas many other p75^NTR^-positive cells lack cdk1 immunolabeling (arrow). (**D**) Cdk1-positive cells co-localize with phosphoHistone H3 in the basal retina, close to the presumptive GCL (arrow). Arrowhead: apically located nucleus with cdk1-specific immunoreactivity. (**E**) TrkB-positive cells co-localize with cdk1 (arrow). Boxes: high magnification of the dashed areas. GCL: ganglion cell layer; PE: pigment epithelium; v: vitreous body. Bar: 20 µm (**A**-**D**), 40 µm (**E**).

We hypothesized that the G2/M arrest induced by BDNF in differentiating RGCs [15] should be mediated through a putative effect of its neurotrophic receptor TrkB on cdk1. Therefore, the precise localization of cdk1 in the E6 chick retina was studied using cdk1-specific antibodies. Cdk1 immunostaining was detected in the cytoplasm of a subset of retinal cells as well as in a minority of nuclei often located at the apical side of the neuroepithelium (Fig. 2A) where mitosis takes place, as expected from the nuclear translocation of cdk1 during prophase [42]. A similar pattern was observed with two alternative antibodies that recognize an additional epitope within the C-terminus of the molecule (Fig. 2B,D). To verify whether cdk1 is expressed in differentiating, p75^NTR^-positive RGCs double immunostaining was performed with antibodies specific for cdk1 and p75^NTR^ in cryosections from E6 chick retinas. This analysis demonstrated that cdk1 is expressed by a subpopulation of p75^NTR^-positive, differentiating RGCs (see arrowhead in Fig. 2C). A quantitative analysis indicated that this subpopulation represents 10.82±2.64% (n = 4) of the p75^NTR^-positive cells, a percentage resembling the proportion of RGCs that reactivate the cell cycle and become tetraploid [15]. As expected, double labeling with an anti-cdk1 antibody along with antibodies recognizing the mitotic marker phosphohistone H3 demonstrated that cdk1 can be observed in basally-located cells undergoing mitosis (Fig. 2D). These cells are known to represent differentiating RGCs that have undergone cell cycle re-entry [15]. Importantly, TrkB immunostaining was detected in all cdk1-positive cells analyzed (98 out of 98 neurons, n = 2 embryos) (Fig. 2E), an observation consistent with the hypothesis that BDNF/TrkB prevents G2/M transition in differentiating neurons through its effects on cdk1.

### BDNF Reduces cdk1 Expression Levels in DCRNs

**Figure 3:**
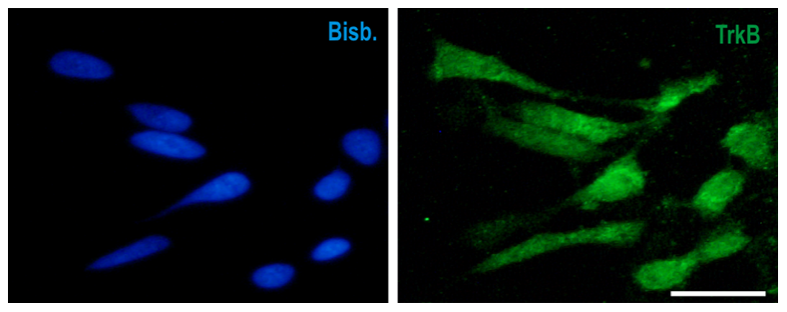
Generalized expression of TrkB in DCRNs. After 20 h in vitro, DCRNs were immunolabeled with anti-TrkB antibody (green) and counterstained with bisbenzimide (Bisb.). Bar: 10 µm.

To determine whether the G2/M arrest induced by BDNF in tetraploid neurons [15] could be explained by a reduction of cdk1 protein expression, we used a previously described in vitro system in which dissociated E6 chick retinal cells are cultured under neurogenic conditions to give rise to DCRNs [12], [15], [17], [33], [34]. DCRNs are known to recapitulate the differentiation of RGCs in vivo. Indeed, these cells express RGC markers [12], [15], [17] as well as E2F1 [15], whereas Rb protein expression is restricted to a specific subpopulation of DCRNs, as occurs in vivo [15]. Moreover, DCRNs can undergo cell cycle re-entry and tetraploidization in response to p75^NTR^ activation by NGF [15], [17], while BDNF has been shown to prevent both mitosis and apoptosis in NGF-treated DCRNs [17]. This latter observation is consistent with the expression of TrkB in all DCRNs (Fig. 3), which facilitates the analysis of the TrkB-dependent signaling described below.

**Figure 4:**
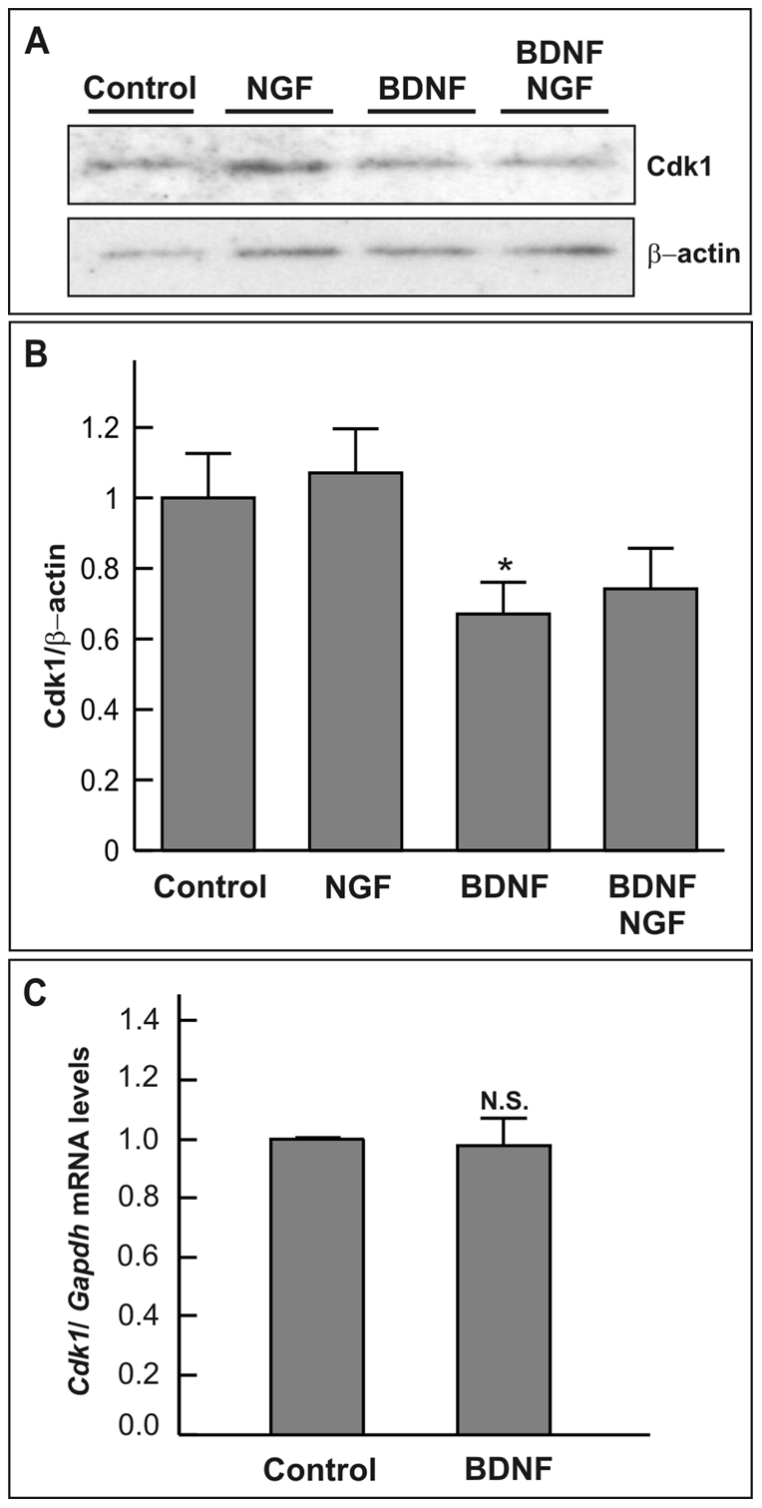
BDNF reduces cdk1 protein levels in DCRNs. (**A**) Lysates from DCRNs cultured for 20 h in the presence of different combinations of 100 ng/m NGF and 2 ng/ml BDNF were subjected to western blot with antibodies specific for either cdk1 (upper panel) or β-actin (lower panel). (**B**) Normalized cdk1/β-actin ratio. (**C**) Semiquantitative RT-PCR analysis of mRNA samples obtained from DCRNs cultured for 20 h in the presence of either vehicle (Control) or 2 ng/ml BDNF (BDNF). The levels of *Cdk1* expression were normalized to *Gapdh*. N.S. non-significant; *p<0.05 (Student’s *t* test; n = 3–4).

To analyze whether BDNF prevents cdk1 expression in DCRNs, extracts from E6 dissociated chick retinal cells, cultured for 20 h under neurogenic conditions with different combinations of NGF (100 ng/ml) and BDNF (2 ng/ml), were analyzed by western blot with antibodies against cdk1. This analysis demonstrated that BDNF is able to significantly reduce the levels of cdk1 in DCRNs (Fig. 4A,B), an effect that was not due to the selective response of a subpopulation of DCRNs to BDNF, as revealed by cdk1-specific immunohistochemistry (data not shown). Semiquantitative RT-PCR performed with mRNA obtained from control and BDNF-treated cultures demonstrated that BDNF did not alter the expression levels of *Cdk1* (Fig. 4C), suggesting that the effect of BDNF on protein levels is post-translational. Interestingly, around 60% of the amount of cdk1 observed in the control situation was still present in BDNF-treated DCRNs. Therefore, G2/M arrest induced by BDNF in DCRNs [17] cannot be fully explained in terms of cdk1 protein downregulation, thus suggesting that additional mechanism should exist for this effect to occur.

### BDNF Reduces cdk1 Specific Activity in DCRNs

**Figure 5:**
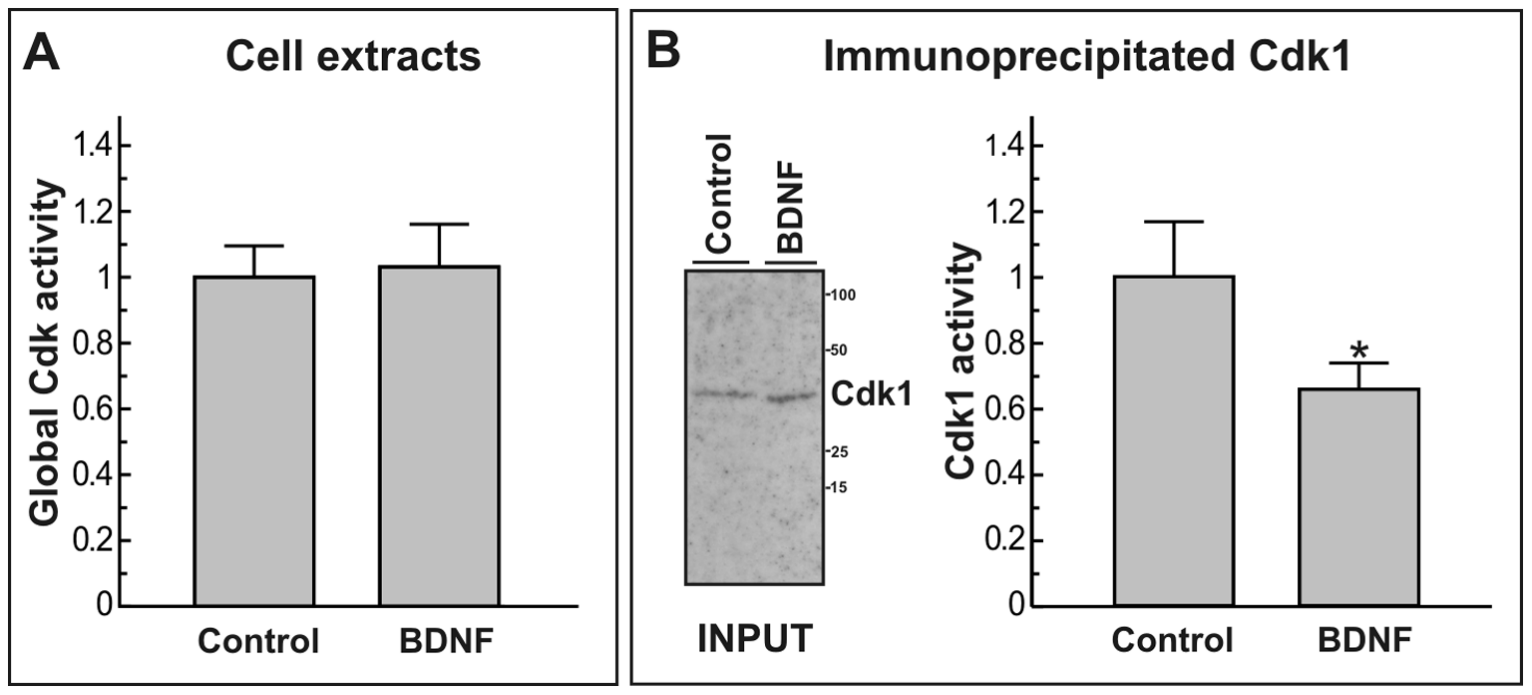
Cdk1 protein kinase activity measured from DCRNs in either the presence or absence of BDNF. (**A**) General cdk protein kinase activity, based on the phosphorylation of a Rb-derived peptide by the cdk activity present in cell extracts from DCRNs cultured for 20 h in the presence of 100 ng/ml NGF and treated for 30 min with either vehicle (Control) or 2 ng/ml BDNF. Equal amount of cell extracts were used in both cases. (**B**) Left panel, a representative western blot performed with an anti-cdk1 antibody using the cell extracts described above prior to immunoprecipitation (INPUT). Right panel, protein kinase activity immunoprecipitated with an anti cdk1 antibody (i.e. cdk1-specific kinase activity) from the cell extracts described above, normalized to the relative amount of cdk1 present in these extracts (see input in left panel). *p<0.05 (ANOVA; n = 3).

The relatively small decrease of cdk1 expression in response to BDNF is likely insufficient to fully prevent G2/M transition in DCRNs. To test whether BDNF can also alter the specific activity of cdk1 in these neurons, an in vitro kinase assay, based on the capacity of cdk1 to phosphorylate in vitro an Rb-specific peptide [40], was performed. To this aim cell extracts from DCRNs cultured for 20 h in the presence of 100 ng/ml NGF to facilitate cell cycle re-entry [17], and treated for 30 min before cell extraction with either 2 ng/ml BDNF or vehicle were used. In this assay, cdk1 was immunoprecipitated with an anti-cdk1 antibody that specifically recognizes this cdk [39] and the kinase activity in the immunoprecipitates was then normalized to the level of cdk1 present in the input (Fig. 5B, left panel). This analysis demonstrated that the addition of BDNF to the retinal cells resulted in a significant reduction of endogenous cdk1 specific activity, already after 30 min of treatment (Fig 5B, right panel). The effect of BDNF was specific for cdk1 since parallel kinase assays performed with non-immunoprecipitated cell extracts, which may also contain other cdks capable to phosphorylate the Rb-specific peptide used for this assay [40], did not result in significant differences of general cdk activity in response to BDNF (Fig. 5A). Overall, these results demonstrate that BDNF not only reduces cdk1 protein expression in NGF-treated DCRNs but also inhibits the specific activity of this kinase.

### Post-translational Regulation of cdk1 by BDNF

**Figure 6:**
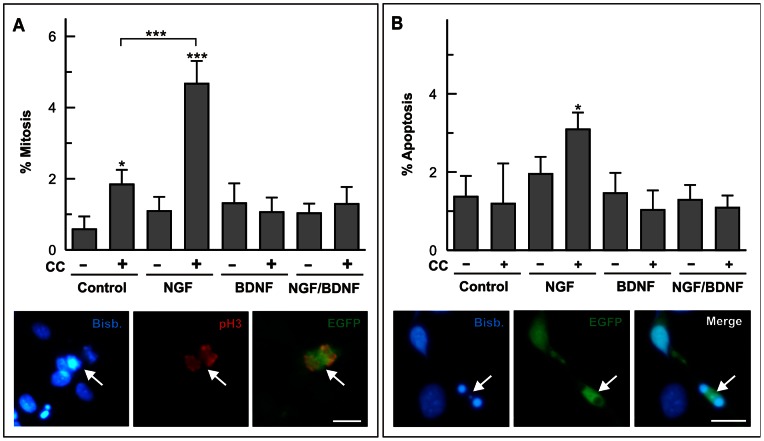
Post-transductional effects of BDNF on cdk1 function. (**A**) E6 retinal cells electroporated with either EGFP (-) or cdk1 plus cyclin B1 (CC) and EGFP (+) were cultured under neurogenic conditions for 20 h in the presence of different combinations of 100 ng/ml NGF and 2 ng/ml BDNF. The percentage of mitotic figures was evaluated in the EGFP-positive cells. Lower panels show an example of a mitotic figure (pH3; red) in an EGFP-transfected cell (EGFP) (arrow). (**B**) E6 retinal cells were electroporated and cultured as above. The percentage of pyknotic nuclei was evaluated in the EGFP-positive cells. Lower panels show an example of a pyknotic nucleus in an EGFP-transfected cell (EGFP) (arrow). Bisb.: bisbenzimide. *p<0.05; ***p<0.005 (Student’s t test; n = 4). Bars: 10 µm.

The decrease of cdk1 specific activity triggered by BDNF in DCRNs could be due to the known inhibitory effect of this neurotrophin on cyclin B1 expression [17]. Nevertheless, additional post-translational mechanisms could also participate in the reduction of cdk1 specific activity by BDNF. To test this hypothesis, E6 retinal cells were co-electroporated with both cdk1 and cyclin B1 pIRES2-EGFP expression vectors, and then cultured for 20 h under neurogenic conditions in the presence of different combinations of 100 ng/ml NGF and 2 ng/ml BDNF. Global analysis of cdk1 activity could not be performed in these cultures since only a small proportion of DCRNs was observed to become electroporated (see Materials and Methods). Therefore, we decided to use the mitotic index of transfected DCRNs as an indirect measure of cdk1 activity in these neurons, since the activation of ectopic cdk1 in cells co-expressing cyclin B1 is expected to increase the proportion of cells in mitosis [43]. This analysis demonstrated that NGF favored the presence of mitotic figures in the transfected cells and that the presence of BDNF dramatically prevented this effect (Fig. 6A). We also analyzed the apoptotic effect of cdk1/cyclinB1 overexpression in DCRNs treated with different combinations of 100 ng/ml NGF and 2 ng/ml BDNF. As expected from previous studies showing that BDNF prevents cell cycle re-entry-dependent cell death in DCRNs [17], BDNF was able to inhibit NGF-dependent cell death in cdk1/cyclin B1-expressing cells (Fig. 6B). Overall, these results indicate that BDNF can modify the activity of cdk1 independently of its capacity to regulate cdk1 and cyclin B1 expression since this neurotrophin was able to induce G2/M arrest and prevent cell death in DNRNs exogenously expressing the cdk1/cyclin B1 complex.

### BDNF Induces Tyr15 Phosphorylation of cdk1, thus Inhibiting its Activity

**Figure 7:**
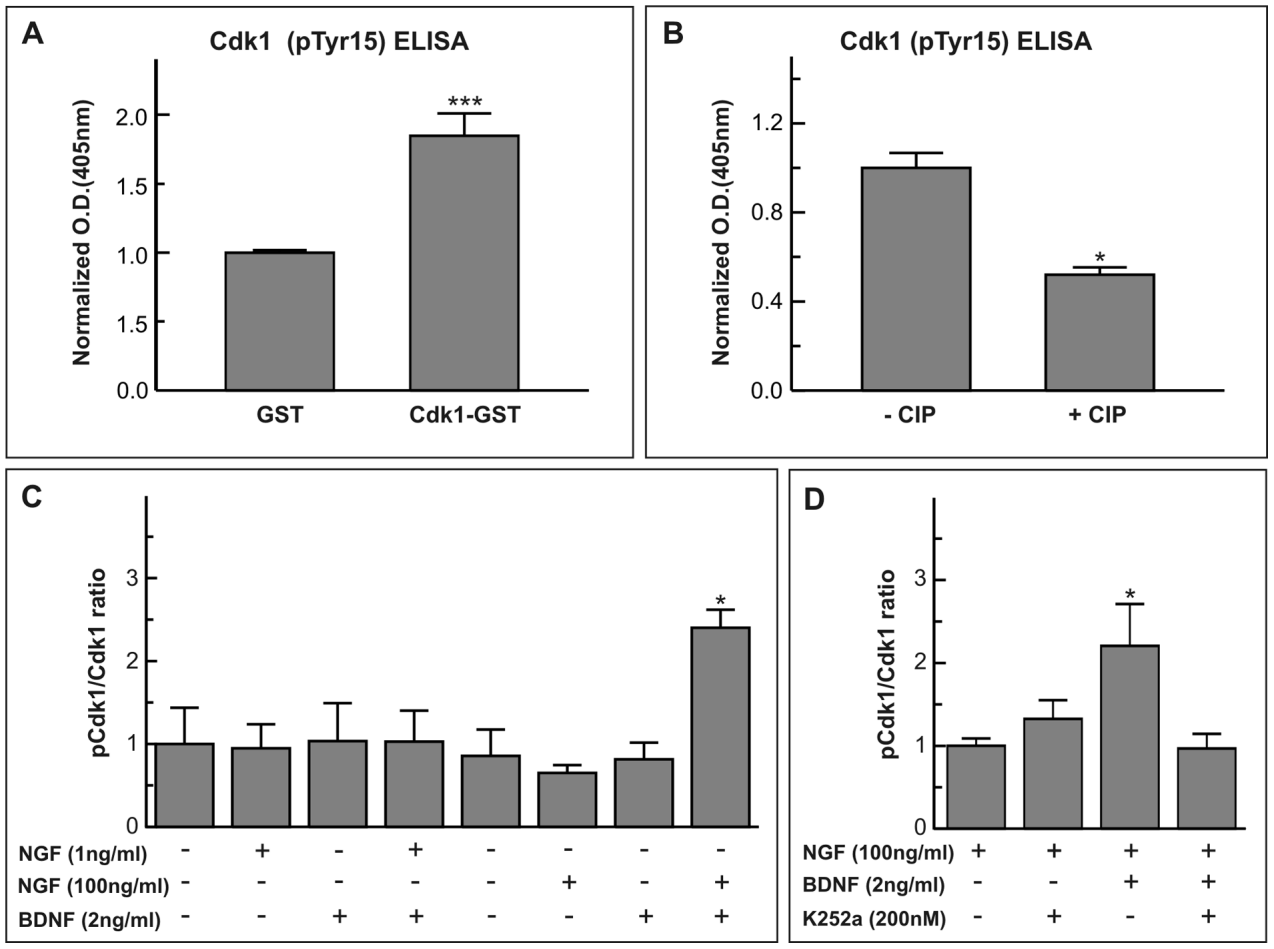
BDNF induces Tyr15 phosphorylation in cdk1. (**A**) Validation of the cdk1 sandwich ELISA assay performed with 10 fg of either GST (GST) or a chimeric protein containing the sequence of human cdk1 bound to GST (Cdk1-GST), and revealed with the anti-cdk1 PSTAIRE antibody. Normalized optical density (O.D.) at 405 nm is shown. (**B**) Validation of the phospho-cdk1 sandwich ELISA assay performed with cell lysates obtained from DCRNs cultured in the presence of 100 ng/ml NGF and 2 ng/ml BDNF, incubated either in the absence (- CIP) or presence (+ CIP) of CIP, and revealed with the anti-cdk1 (pTyr15) antibody. Normalized optical density (O.D.) at 405 nm is shown. (**C**) Cell lysates from DCRNs cultured in the presence (+) or absence (-) of the referred factors were subjected to sandwich ELISA analyses using antibodies specific for either cdk1 phosphorylated at Tyr15 or total cdk1 protein. Colorimetric values for cdk1 phosphorylated at Tyr15 were normalized to the levels of cdk1 protein (Cdk1). (**D**) Cell lysates from DCRNs cultured in the presence of the referred factors and/or K252a were subjected to sandwich ELISA analyses as described above. Colorimetric values for cdk1 phosphorylated at Tyr15 were normalized to the levels of cdk1 protein (Cdk1). *p<0.05; ***p<0.005 (Student’s t test; n = 3).

To decipher the molecular mechanism used by BDNF to prevent cdk1 activity in NGF-treated DCRNs, we became inspired by a previous study showing that TrkB activation leads to cdk5 phosphorylation in Tyr15 [44]. Cdk5, a member of the cdk family that lacks capacity to regulate the cell cycle, becomes activated upon Tyr15 phosphorylation [44]. In contrast, phosphorylation of cdk1 at Tyr15 is known to functionally inhibit its kinase activity, thus preventing G2/M transition [43]. We therefore hypothesized that BDNF, through its TrkB receptor, could lead to Tyr15 phosphorylation of cdk1, thus inhibiting its kinase activity. Western blot cannot be used for unambiguous quantification of phospho-cdk1 levels in cell extracts since the sequence of cdk1 flanking Tyr15 is conserved in cdk2, and the anti-phospho-cdk1 antibodies cannot discriminate between phospho-cdk1 and phospho-cdk2. Immunoprecipitation of cdk1 with a specific antibody, followed by western blot using an anti-phospho-cdk1 cannot be performed either. Indeed, two different antibodies against phospho-cdk1 were observed to detect intense, unspecific bands of molecular weight similar to cdk1 in three different sources of Protein A/G beads (data not shown). Therefore, the hypothesis that BDNF leads to Tyr15 phosphorylation of cdk1 was tested using sandwich ELISA. This assay, able to quantify the amount of both total cdk1 and Tyr15-phosphorylated cdk1, was performed in cell lysates derived from DCRNs maintained for 20 h in the presence of either 100 ng/ml NGF or vehicle, and treated with either 2 ng/ml BDNF or vehicle 30 min before cell extraction. Two different antibodies were used for the sandwich ELISA assay, an anti-cdk1-specific antibody employed for coating and another antibody recognizing the phospho-Tyr15 site of cdk1. As a control, the sandwich ELISA assay was able to specifically detect as low as 10 fg of cdk1-GST chimeric protein, as compared to 10 fg of GST (Fig. 7A). Moreover, our sandwich ELISA assay can detect cdk1 phosphorylated in Tyr15 since the signal obtained with the anti-cdk1 antibody recognizing phosphoTyr15 was significantly reduced in cell lysates incubated with CIP as compared with control cell lysates (Fig. 7B). The sandwich ELISA assay demonstrated that the addition of 2 ng/ml BDNF to DCRNs previously treated with 100 ng/ml NGF significantly increased the levels of phosphoTyr15 in cdk1 (Fig. 7C), thus explaining the capacity of BDNF to trigger G2/M arrest in NGF-treated DCRNs. The effect of BDNF on cdk1 phosphorylation was regulated by TrkB since K252a, a protein kinase inhibitor known to prevent TrkB activity [45], [46], was able to inhibit BDNF-dependent phosphorylation of cdk1 at Tyr15 (Fig. 7D).

Interestingly, in the presence of NGF at 1 ng/ml, BDNF was not able to induce cdk1 Tyr15 phosphorylation (Fig. 7C). Although NGF can induce TrkA-dependent biological effects at 1 ng/ml [37], this latter concentration is below the half maximal effect of NGF to induce p75^NTR^-dependent cell cycle re-entry in DCRNs [17]. We conclude therefore that the effect of BDNF on cdk1 phosphorylation does not require a previous activation of TrkA by NGF. In contrast, it seems that p75^NTR^ needs to be efficiently activated by NGF to allow the effect of BDNF on cdk1 phosphorylation.

### Tyr15 Phosphorylation of cdk1 by BDNF Uses a Mechanism Independent of Wee1

**Figure 8:**
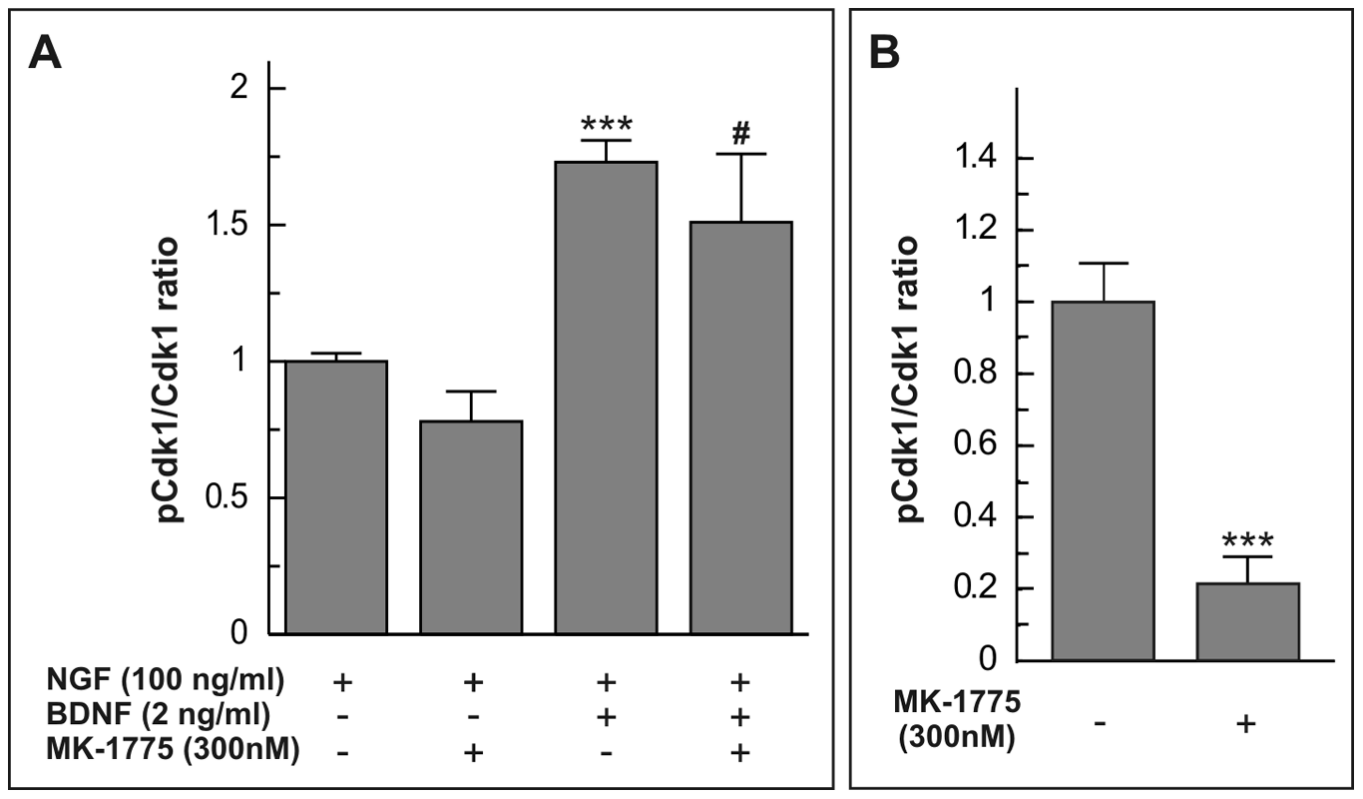
Tyr15 phosphorylation in cdk1 by BDNF is independent of Wee1. (**A**) Cell lysates from DCRNs cultured in the presence of the referred factors and/or 300 nM MK-1775 were subjected to sandwich ELISA analyses using antibodies specific for either cdk1 phosphorylated at Tyr15 or total cdk1 protein. Colorimetric values for cdk1 phosphorylated at Tyr15 were normalized to the levels of cdk1 protein (Cdk1). (**B**) Cell lysates from CEFs cultured in the presence (+) or absence (-) of 300 nM MK-1775 were subjected to sandwich ELISA analyses as described above. Colorimetric values for cdk1 phosphorylated at Tyr15 were normalized to the levels of cdk1 protein (Cdk1). ***p<0.005 (Student’s t test; n = 3). **^#^**p<0.05 (NGF/BDNF/MK-1775 vs NGF/MK-1775; Student’s t test; n = 3).

BDNF-dependent cdk1 phosphorylation in DCRNs was observed to be independent of Wee1, the kinase that phosphorylates cdk1 at Tyr15 during G2 in proliferating cells [47]. In this regard, treatment of DCRNs with 300 nM MK-1775, a potent and selective Wee1 inhibitor known to prevent Wee1 activity when used at this latter concentration [48], could not prevent the effect of BDNF on Tyr15 phosphorylation of cdk1 (Fig. 8A). In contrast, CEFs treated with 300 nM MK-1775 showed an 80% decrease in the level of cdk1 phosphorylation at Tyr15 as compared to the control situation (Fig. 8B). These results indicate that the Wee1 inhibitor can prevent Tyr15 phosphorylation of cdk1 in proliferating cells but not in BDNF-treated DCRNs.

### BDNF cannot Prevent the Apoptotic Effect of a Constitutively Active form of cdk1 on DCRNs

**Figure 9:**
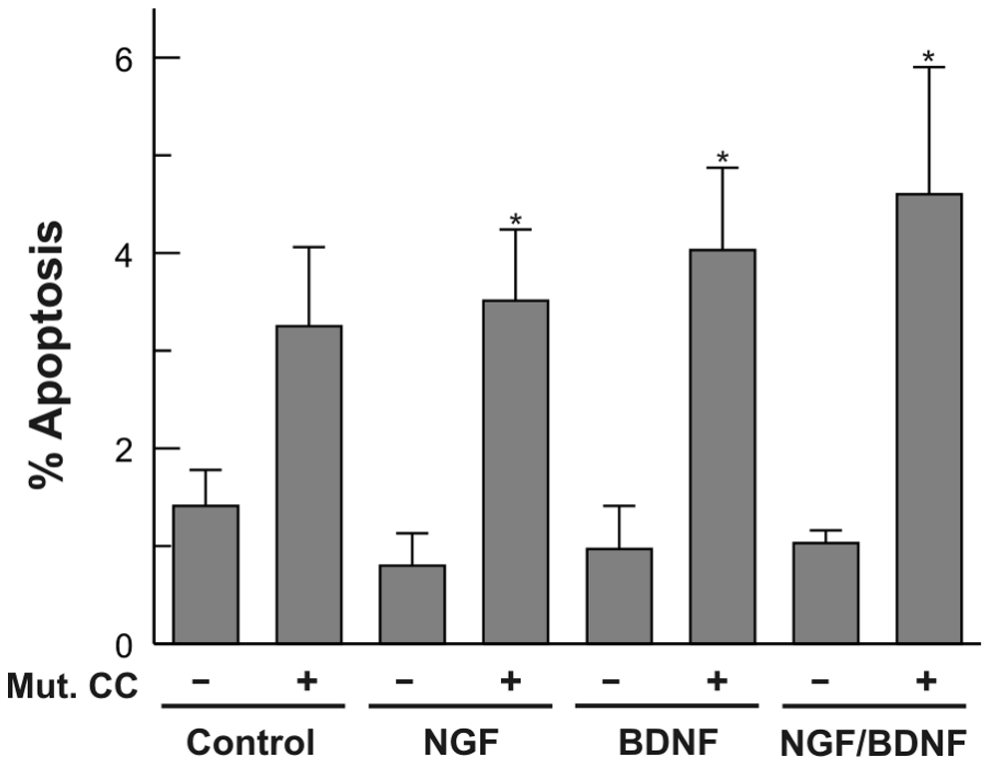
Post-transductional effects of BDNF on cdk1 function are specific for Tyr15. E6 retinal cells electroporated with either EGFP (-) or the Tyr15Phe mutant form of cdk1 plus cyclin B1 (Mut CC) and EGFP (+) were cultured for 20 h under neurogenic conditions in the presence of different combinations of 100 ng/ml NGF and 2 ng/m BDNF. The percentage of pyknotic nuclei was evaluated in EGFP-positive cells. *p<0.05 (Student’s t test; n = 4).

To test whether BDNF could use additional mechanisms to prevent cdk1 activity, E6 retinal cells were co-electroporated with two pIRES2-EGFP vectors expressing both cyclin B1 and a constitutively active form of cdk1 in which Tyr15 has been substituted by Phe (Tyr15Phe) [32]. Then, E6 retinal cells were cultured for 20 h under neurogenic conditions in the presence of different combinations of 100 ng/ml NGF and 2 ng/ml BDNF. Overexpression of the mutated form of cdk1 was able to induce ectopic mitoses followed by cell death. The percentage of detectable mitoses after 20 h of treatment was very small (<1%), suggesting that the presence of an active form of cdk1 may accelerate the mitotic process. This notion was confirmed when G2/M transition was blocked with nocodazole, a known inhibitor of the microtubule polymerization. Application of 0.4 µg/ml nocodazole for 18 h resulted in the presence of phosphoHistone H3 in a significant number of DCRNs previously transfected with the mutated form of cdk1 and maintained in the presence of 100 ng/ml NGF (11 out of 110 cells analyzed), as well as low levels of apoptosis, measured as the percentage of cells showing pyknotic nuclei (2 out of 110 cells analyzed). In contrast, apoptosis was readily observed in the transfected cells cultured in the absence of nocodazole (Fig. 9). Moreover, the presence of the Tyr15Phe mutation inhibited the effect of BDNF in preventing cdk1/cyclin B1-dependent apoptosis (Fig. 9). We concluded therefore that BDNF inhibits cdk1 function specifically through phosphorylation of Tyr15.

## Discussion

**Figure 10:**
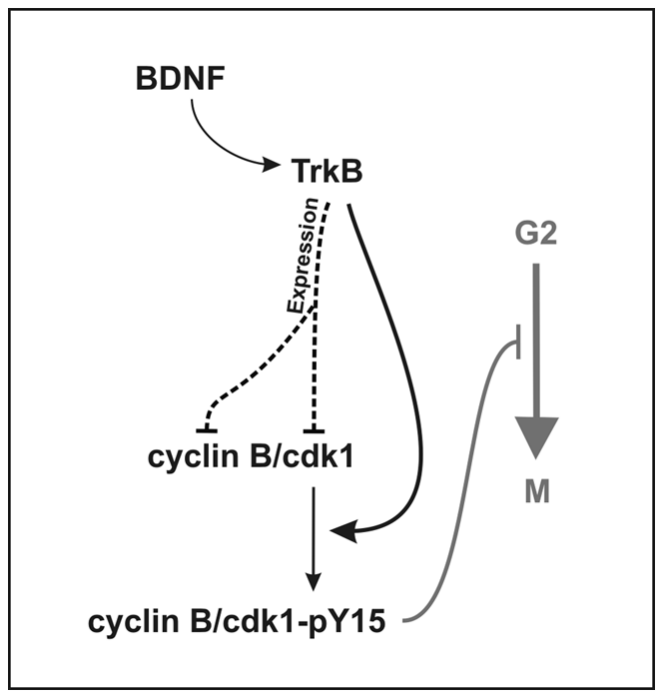
A model for the regulation of cdk1 by BDNF in DCRNs. TrkB activation by BDNF prevents the increase cyclin B and cdk1 protein expression (dashed line) and induces phosphorylation of cdk1 at Tyr15 (thick black arrow), thus inhibiting cdk1 kinase activity. This effect participates in the G2/M arrest (grey line) observed in tetraploid RGCs.

In this study we have described two mechanisms used by BDNF to prevent G2/M transition in tetraploid neurons, which rely on the reduction of cdk1 (this study) and cyclin B1 [17] expression, as well as the induction of Tyr15 phosphorylation in cdk1, thus inhibiting its kinase activity (Fig. 10).

We have demonstrated the presence of cdk1 in cells that undergo ectopic mitosis at the basal portion of the retina, previously identified as differentiating RGCs that have reactivated the cell cycle and are about to undergo apoptosis [15], indicating that the common mechanism for G2/M transition can be active in these cells. This observation is consistent with a previous study in the quail showing cdk1 expression in postmitotic RGCs before they are fully differentiated [49]. We provide two lines of evidence demonstrating that cdk1 function can be regulated by BDNF in tetraploid neurons. Firstly, we demonstrate that BDNF reduces the levels of cdk1 protein, but not of *Cdk1* mRNA, in DCRNs, thus suggesting that BDNF uses a post-translational mechanism for this regulation. Although the expression of cdk1 has been shown to be downregulated as the retina differentiates [49], the effect of BDNF on cdk1 expression is likely specific since this neurotrophin cannot induce neuronal differentiation in retinal precursors [50]. Secondly, using a kinase assay we have shown that BDNF is also able to reduce cdk1 specific activity in DCRNs-derived extracts. This reduction of the specific activity of cdk1 cannot simply be explained by the known decrease of cyclin B2 expression triggered by BDNF in NGF-treated DCRNs [17], since BDNF was able to prevent cdk1 activity and apoptosis in NGF-treated DCRNs that over-express both cdk1 and cyclin B1. The regulation by BDNF of cdk1 expression and function in tetraploid neurons is reminiscent from the effect of NGF on this cdk during NGF-dependent PC12 cell differentiation, which leads to the reduction of the cdk1 protein levels and a decrease in its enzymatic activity, an effect accelerated in cells that over-express TrkA [51].

Most of this study has been performed with embryonic retinal cells cultured under neurogenic conditions [33], [34], and treated with NGF to induce cell cycle re-entry [12], [15], [17]. In these cultures, BDNF is likely acting through the neurotrophic receptor TrkB, which is expressed by all DCRNs. Indeed, picomolar concentrations of BDNF (i.e. 2 ng/ml), known to be specific for its high-affinity receptor [38], can induce G2/M arrest in NGF-treated DCRNs. Moreover, the use of K252a, a known inhibitor of protein kinases commonly used to prevent the activation by neurotrophins of Trk receptors including TrkB [45], [46], was able to prevent BDNF-dependent phosphorylation of cdk1 at Tyr15. As in the DCRNs, BDNF is also likely to induce TrkB-dependent G2/M arrest in tetraploid RGCs in vivo since BDNF is expressed in the pigment epithelium and retina during the period of RGC differentiation [16], and we have shown TrkB immunoreactivity in differentiating RGCs lacking Rb expression, known to become tetraploid in response to endogenous NGF [15].

The mechanism used by BDNF to directly inhibit the activity of cdk1 relies on Tyr15 phosphorylation of this cdk. This conclusion is consistent with the observation that BDNF cannot prevent in DCRNs the apoptotic effect of a constitutively active form of cdk1 in which Tyr15 has been substituted by a Phe residue. In proliferating cells, cdk1 is phosphorylated in Tyr15 by the G2/M check point kinase Wee1 [47], thus preventing its biological activity [43]. Interestingly, Wee1 has been shown to be expressed by differentiating neurons, playing an essential role for the initial differentiation prior to axonal polarization [52], and also by adult neurons [53]. In agreement with a cell cycle-independent role of Wee1 in differentiating neurons we have shown that a selective inhibitor of this latter kinase, used at a concentration previously shown to block Wee1 activity [48], was unable to inhibit the effect of BDNF on cdk1 phosphorylation. Based on the above evidence, we can rule out the participation of Wee1 in the signaling pathway initiated by TrkB to arrest tetraploid neurons in G2. TrkB might regulate cdk1 activity by either direct interaction followed by Tyr15 phosphorylation, as it has been shown for cdk5 [44], or by cdk1 phosphorylation by other protein kinases acting downstream of TrkB. In this latter case, it is noteworthy that cdk1 can be inactivated by Tyr15 phosphorylation in an ERK-dependent manner [54], the latter being a major pathway activated by Trk receptors [55].

Neurotrophin signaling has proven to be highly complex due to the existence of different receptors with intricate functionality [24]. There is published evidence that Trk receptor-dependent signaling can be modulated by p75^NTR^ activation with heterologous ligands. For instance, BDNF binding to p75^NTR^ has been shown to modulate both NGF/TrkA- and NT3/TrkC-dependent signaling [56]–[58]. A similar situation is observed in our study. We have demonstrated that TrkB-dependent phosphorylation, but not expression regulation, of cdk1 in DRCNs required previous activation of p75^NTR^ by NGF. This observation suggests that the levels of cdk1 expression in DCRNs are not modulated by Tyr15 phosphorylation. BDNF-dependent phosphorylation of cdk1 at Tyr15 does not seem to require TrkA activity since a p75^NTR^ suboptimal concentration of NGF (1 ng/ml) was unable to facilitate the effect of BDNF in Tyr15 cdk1 phosphorylation. This latter observation is consistent with the capacity of a p75^NTR^ blocking antibody to prevent NGF-dependent cell cycle re-entry in DCRNs [17]. The mechanism used by p75^NTR^ to facilitate the phosphorylation of cdk1 at Tyr15 is currently unknown and further studies will be necessary to fully characterize it.

Cdk1 expression has been shown to be downregulated at E8 in the developing quail retina [49], a stage equivalent to E10–11 in the chick embryo [59]. At this stage, chick RGCs generation has been completed [60], and the number of high-affinity binding sites for BDNF is dramatically reduced by this age in the chick retina [16]. Altogether, these observations indicate that the mechanism described in this study for BDNF-dependent inhibition of cdk1, required for tetraploid neuron survival during development, seems to be restricted to a specific temporal window just when RGCs differentiate.

BDNF-dependent G2/M arrest in tetraploid neurons has been shown to prevent cell death [15], [17]. Likewise, mitotic arrest with nocodazole reduced the levels of cell death in DCRNs transfected with a constitutively active form of cdk1. These observations suggest that cdk1 activity may participate in the induction of apoptosis in these neurons. In this regard, neuronal cdk1 activation can lead to nuclear accumulation of FOXO1 and the phosphorylation of Bad, thus triggering apoptosis [7], [61]. The hypothetical prevention of neuronal apoptosis by BDNF through inactivation of cdk1 might be a crucial mechanism for the maintenance of tetraploid neurons during both embryonic development and also in the normal and pathological adult brain. In this regard, active cdk1/cyclin B1 kinase has been found to be enriched in AD neurons with neurofibrillary tangles [62]–[65], and BDNF is known to facilitate neuronal survival in a number of neurodegenerative disorders [66]. The known decrease of BDNF and TrkB in the brain at late stages of AD [67] could therefore facilitate neuronal degeneration [22].

In sum, in this study we have provided evidence for a novel mechanism likely based on the activity of the TrkB neurotrophic receptor, which regulates the maintenance of vertebrate tetraploid neurons in a G2-like state, thus preventing their death. This mechanism might be active in neurodegenerative states in which neuronal tetraploidization is known to occur.
